# Supplementary material for: Dietary Intake of Adolescents and Alignment with Recommendations for Healthy and Sustainable Diets: Results of the SI.Menu Study
Source: Nutrients. 2024 Jun 17;16(12):1912. doi: 10.3390/nu16121912 (PMC11206795; doi:10.3390/nu16121912)
Supplement: Supplementary file 1 [file nutrients-16-01912-s001.zip › nutrients-3025903-supplementary.pdf]

Supplementary Table S1: Food Groups

**Food Groups**	**Food and beverages groups description-included food items**
Milk	Whole milk, semi-skimmed and skimmed milks, full-fat milk, pasteurised whole milk, fresh milk, long-lasting sterilized milk, lactose free milk, chocolate whole and semi-skimmed milk, milk with added vitamins and minerals, condensed milks and evaporated milks, others …
Dairy products	Chilled dairy products, cream, yoghurt, yoghurt, flavoured yoghurt, pudding, crème fraiche, plain curd (quark), curd with herbs, quark dessert, cottage cheese and petit-suisse, yogurt, milk cream, fruit yogurt, Greek yogurt, sour milk, buttermilk, kefir, cottage cheese, quark, all types of cheese - mozzarella, feta, semi-hard and hard cheese, others ...
Cheese	All types of cheese - mozzarella, ricotta, kajmak, feta, semi-hard and hard cheese, emmentaler, gouda, trapist, edamer, edam, tilsit, cheddar, tolminc, zbrinc, parmesan, pecorino, firm cheese, hard, stretched or brined cheeses, soft cheeses with moulded rind, soft cheeses with washed rind, blue cheeses, processed cheeses, uncured cheeses, others ...
Vegetables	Fresh and preserved/canned vegetables
Fresh vegetables	Peppers, tomatoes, cucumbers, pickles, zucchini, eggplants, lettuce, spinach, Swiss chard, kohlrabi, carrots, broccoli, cauliflower, white cabbage, kale, Brussels sprouts, red cabbage, sauerkraut, olives, avocado, asparagus, mixed vegetables, onion, garlic, and others ...
Preserved and canned vegetables	Cooked, boiled, drained beets, pickled, canned, solid and liquid cabbage, cooked, boiled, drained, peppers, hot chili, green, canned, pods, excluding seeds, pickles, cucumber, cooked, boiled, drained, without salt salsify, cooked, boiled, drained, without salt sauer turnip, sauerkraut, canned tomatoes, red, ripe, canned, packed in tomato juice and others ...
Fruits	Fresh, canned, dry fruits.
Fresh fruits	Apple, pear, orange, tangerine, kiwi, cherry, plum, peach, apricot, nectarine, pomegranate, grape, melon, banana, strawberry, blueberry, raspberry, gooseberry, fresh fig, blueberry, fruit salad and others.
Other fruits (canned, dry)	Pickled fruit (compote), dried fruit, candied fruit, mashed and cooked fruit …
Legumes	Kidney beans, soybean, mung bean, lima beans, peas, lentils, chickpeas, pulses broad beans, haricot beans, split peas, tofu …
Nuts and seeds	Nuts, seeds, flax seed, trail mix, other …
Potatoes	Potatoes, pan-fried potatoes, mashed potatoes, potato dumplings, potato salad, filled potatoes, potato pancake, chips/French fries, sweet potato, frozen potatoes, chips, potato crisps, and other.
Bread and bakery products	All type of breads, rusk and other bread products, bread dough …
Bread	Wheat bread, baguette, bread crumbs, toast, mix grain breads, small bread - kajcar, corn mixed bread, ray mixed bread, oat mixed bread, wholegrain bread, French bread, pita bread, loaf bread, panini bread …
Cereal and cereal products	Breakfast cereals, pasta, rice, semolina and wheat, muesli, corn flakes, wholemeal, rolled cereals/oat flakes, puffed rice, other cereals, crisps and crackers, etc.
Breakfast cereals	Corn flakes, sweet flakes, čokolino, sport muesli, wholemeal, rolled cereals/oat flakes, puffed rice, other cereals …
Pasta, rice	Pasta, rice, wheat brown and white rice, noodles, whole grain pasta, groats and others.
Fish and fish products	Raw fish, canned and cooked fish and fish-based products, salmon, mackerel, herring, herring salad, salted herring, fried herring, rolled pickled herring, hot smoked herring, sprat, eel, redfish, trout, tuna, saithe/pollock, codfish, fish sticks, fish bake, calamari, craps/shellfish and other.
Fresh fish	Raw fish, freshwater and saltwater fish.
Fresh meat	Red meat and poultry.
Red meat	Lamb, beef, veal and pork meats, offal brain, heart, liver, tongue, sweetbreads and kidneys, minced meat, venison, mixed ground meat, beef roulade, beef goulash, roast pork, pork goulash, gyros, shashlik/meat skewer, hamburger/meatball …
Poultry	Poultry and game chicken, turkey, duck meats and game.
Processed meat	Meat products raw and cooked hams, sausages, salami, ham, prosciutto, bacon, pâté's and rillettes (potted meat), liverwurst, mettwurst, cabanossi, bologna/baloney, ham sausage, cooked ham, raw ham, poultry sausage, aspic, collared pork, blood sausage, frankfurter/wiener/hot dog, bratwurst, veal sausage, meat loaf, meat salad, and other processed meat.
Fruit and vegetable juices	Fruit and vegetable juices, nectars, multi-vitamin juice, apple juice, orange juice, grape juice, grapefruit juice, elder juice, lemon juice, other fruit juice, tomato juice, other vegetable juice …
Sugar-containing soft beverages	Cold sweetened beverages, fruit-based sweeten beverages, lemonade, diet lemonade, cola, diet cola, non-alcoholic beer, malt beer, other.
Tap and bottled water	Plain water, carbonated mineral waters, tap water, spring water.

Hot drinks	Coffee, tea, cacao and hot chocolate, other hot drinks or chocolate beverages, chicory coffee, tea and herbal tea.
Tea	Fruit and herbal tea, black tea, green tea.
Coffee drinks	Coffee/espresso, coffee without caffeine, cappuccino …
Fats and oils	Vegetable oils (olive oil, vegetable oil, wheat germ oil, safflower oil, sunflower oil, grape seed oil, linseed oil, rapeseed oil, other), margarines, butter and other animal fat.
Vegetable oils and fats	Margarines, olive, soybean, pumpkin, sunflower, flaxseed, coconut, corn, canola and palm oil.
Butter and other animal fat	Animal fat, bacon grease, lard, homemade minced lard.
High sugar food	Sugar, confectionary, cakes, cookies, desserts.
Sugar and confectionary	Sugar and confectionery, marmalades, jams, honey and non-chocolate confectionery, chocolate, chocolate candy bars and chocolate hazelnut spread, sugar beet molasses, chocolate mousse, tiramisu, cold sweet soup with fruit, red fruit jelly, filled chocolates, chocolate bar, other chocolate or sweets with chocolate, other sweets without chocolate, sugar as addition.
Cakes, cookies	Sweetened pastries and biscuits, brioche, croissants, croissants with chocolate filling, milk bread rolls, cakes, pastries, sweet pies, fritters, crêpes, waffles and preparations for pastries, shortbread biscuits, chocolate biscuits, flaky pastry, biscuits with fruit or jam filling, butter biscuits, wafers, meringues, cookies ...
Desserts	Cream desserts and milk jellies custard desserts and jellified milks, ice cream, frozen desserts and sorbets.
Eggs	Eggs and egg products fried, scrambled, raw, hard-boiled and poached eggs, omelettes.
Ready to eat meals and dishes	Mixed dishes, soups and stocks ready-to-eat, instant soups and stocks, vegetable-based dishes, side dishes, vegetable stew, stew with meat, clear soup, crème of vegetable soup, other. The foods for which the recipes were not available – usually store-bought ready to eat meals.

Supplementary Table S2: True non-consumers, means of estimated individual consumption probabilities and frequencies, person-specific daily consumption amounts (gram/day), and individual usual food intakes (gram/day) for adolescent males and females (10–17 years of age).

Food group	Male adolescents (n = 238)					Female adolescents (n = 230)				
	True non-consumers N (%) #	Consumption probability*	Mean consumption frequency (ff/day) $	Amount	Usual intake	True non-consumers N (%)*	Consumption probability*	Mean consumption frequency (ff/day) $	Amount	Usual Intake
Milk	6 (2.5)	0.80	0.71	202.4	161.6	10 (4.3)	0.68	0.65	173.3	117.5
Dairy products	3 (1.3)	0.78	0.82	85.3	66.7	2 (0.9)	0.73	0.79	84.4	62.0
Cheese	12 (5.0)	0.72	0.41	46.3	33.5	7 (3.0)	0.68	0.37	43.8	29.9
Vegetables	1 (0.4)	1.00	0.68	110.1	110.1	1 (0.4)	1.00	0.72	107.5	107.0
Fresh vegetables	1 (0.4)	1.00	0.58	81.8	81.8	1 (0.4)	1.00	0.63	84.0	83.6
Preserved and canned vegetables	26 (11.0)	0.68	0.15	39.3	26.6	34 (14.8)	0.58	0.14	35.3	20.5
Fruits	3 (1.3)	0.48	0.88	319.5	154.3	1 (0.4)	0.46	0.92	291.9	132.9
Fresh fruits	3 (1.3)	0.71	0.70	194.4	138.2	1 (0.4)	0.78	0.75	197.5	154.4
Other fruits (canned, dry)	35 (14.7)	0.13	0.21	112.7	14.4	73 (31.7)	0.19	0.20	90.6	17.0
Legumes	32 (13.4)	0.17	0.23	57.7	9.9	45 (19.6)	0.19	0.21	54.4	10.2
Nuts and seeds	79 (33.2)	0.16	0.14	42.7	7.0	68 (29.6)**	0.15	0.15	54.2	8.0
Potatoes	0 (0.0)	0.70	0.54	136.7	95.4	0 (0.0)	0.73	0.56	114.5	83.6
Bread and bakery products	0 (0.0)	1.00	1.23	217.3	216.4	0 (0.0)	0.97	1.16	160.4	154.8
Bread	0 (0.0)	0.99	1.05	165.1	163.0	0 (0.0)	0.94	1.00	123.6	116.1
Cereal and cereal products	0 (0.0)	0.94	0.87	154.7	145.6	0 (0.0)	0.94	0.84	123.5	116.0
Breakfast cereals	11 (4.6)	0.82	0.42	88.0	71.8	3 (1.3)	0.79	0.39	73.0	57.8
Pasta, rice	0 (0.0)	0.70	0.48	136.7	95.4	0 (0.0)	0.73	0.45	114.5	83.6
Fish and fish products	18 (7.6)	0.21	0.18	105.6	22.2	25 (10,9)	0.22	0.17	84.6	18.8
Fresh fish	47 (19.7)	0.09	0.08	132.2	12.2	60 (26,9)	0.08	0.09	130.3	10.2
Fresh meat	0 (0.0)	1.00	0.66	169.7	169.0	3 (1.3)	0.97	0.59	135.2	131.5
Red meat	0 (0.0)	0.94	0.33	101.6	95.2	8 (3.5)	0.87	0.28	76.0	65.8
Poultry	0 (0.0)	0.67	0.36	111.1	74.1	8 (3.5)	0.69	0.36	95.8	66.1
Processed meat	5 (2.1)	0.45	0.28	107.9	48.9	17 (7.4)	0.31	0.23	87.4	27.4
Fruit and vegetable juices	29 (12.2)	0.47	0.27	256.8	121.9	22 (9.6)	0.48	0.28	232.6	111.3
Sugar-containing soft beverages	5 (2.1)	0.24	0.50	427.1	101.3	16 (7.0)	0.20	0.44	393.0	79.0
Hot drinks	14 (5.9)	0.84	0.47	346.6	290.8	10 (4.3)	0.82	0.58	337.2	277.3
Tea	15 (6.3)	0.51	0.41	328.9	168.8	14 (6.9)	0.46	0.50	329.0	152.3
Coffee drinks	185 (77.7)	0.81	0.33	153.4	123.8	169 (73.5)	0.81	0.36	143.3	116.1

Fats and oils	0 (0.0)	1.00	1.27	22.3	22.3	0 (0.0)	1.00	1.42	17.9	17.9
Vegetable oils and fats	1 (0.4)	0.99	0.94	16.0	15.9	1 (0.4)	0.99	1.04	13.7	13.5
Butter and other animal fat	16 (6.7)	0.39	0.40	17.2	6.7	10 (4.3)	0.34	0.42	12.8	4.4
High sugar food	0 (0.0)	0.90	1.60	139.2	125.6	0 (0.0)	0.89	1.76	141.6	125.6
Sugar and confectionary	0 (0.0)	0.69	1.00	50.7	35.0	0 (0.0)	0.74	1.16	42.2	31.3
Cakes, cookies	21 (8.8)	0.36	0.28	101.7	36.3	24 (10.4)	0.36	0.32	88.0	31.8
Desserts	0 (0.0)	0.35	0.30	194.9	68.0	0 (0.0)	0.40	0.30	154.5	62.5
Eggs ***	138 (58.0)				37.1	127 (55.2)				33.1
Ready to eat meals	70 (29.4)	0.54	0.15	83.8	44.9	69 (30.0)	0.51	0.09	72.0	36.7

Note: Usual intake is described with the mean of all respondents usual food groups intakes. # Number of consumers of a given food group identified in a probabilistic model by zero frequency of consumption as well as no reported consumed quantities. * Consumption probability describes the percentage of the daily habitual consumers in the sex population. $ Long-term consumption frequency information from a food propensity questionnaire. The average frequency equal to 1 means frequency of consumption of food group on daily basis; the average frequency more than 1 describe frequency of consumption exciding consumption of one time a day; the average frequency of less than 1 means infrequent consumption which is less than once a day. *** Data is provided without correction for usual intake and describe habitual two-day average consumption.

Supplementary Table S3: Selection of determinants for daily consumption intakes across 18 food groups for adolescents aged 10–17 years.

**Food group**	Cohesion regions	Sex	Age groups	BMI	Place of residence	IPAQ (self-assessed)	Household composition	Season
Sugar and confectionary	X		X	X	X	X	X	X
Animal fats		X	X	X	X	X		X
Bread and bakery products (all type of breads, dough, bread based products)	X	X					X	X
Pasta and rice	X	X			X	X		X
Red meat		X	X	X				X
Milk	X			X				X
Fats and oils (vegetable oils, margarines, butter and other animal fat)		X			X			X
Vegetable oils		X	X					X
Fruits	X		X			X		
Bread		X	X			X		
High sugar food (sugar, confectionary, cakes, cookies, desserts)	X			X				X
Poultry				X	X			
Vegetables (fresh and preserved/canned)	X							X
Legumes					X			X
Dairy products	X			X				
Process meat		X				X		
Fish and fish products			X		X			
Cereal and cereal products (breakfast cereals, pasta, rice, etc.)		X						

Note: X sign denotes determinant with significant influence on the food group consumption.
